# Pathogenic *FAM83G* palmoplantar keratoderma mutations inhibit the PAWS1:CK1α association and attenuate Wnt signalling.

**DOI:** 10.12688/wellcomeopenres.15403.2

**Published:** 2020-02-17

**Authors:** Kevin Z.L. Wu, Rebecca A. Jones, Theresa Tachie-Menson, Thomas J. Macartney, Nicola T. Wood, Joby Varghese, Robert Gourlay, Renata F. Soares, James C. Smith, Gopal P. Sapkota

**Affiliations:** 1 Medical Research Council, Protein Phosphorylation and Ubiquitylation Unit, University of Dundee, Dundee, UK; 2 The Francis Crick Institute, London, UK

**Keywords:** Wnt signalling, palmoplantar keratoderma, casein kinase, skin, hereditary footpad hyperkeratosis.

## Abstract

**Background:** Two recessive mutations in the *FAM83G* gene, causing A34E and R52P amino acid substitutions in the DUF1669 domain of the PAWS1 protein, are associated with palmoplantar keratoderma (PPK) in humans and dogs respectively. We have previously reported that PAWS1 associates with the Ser/Thr protein kinase CK1α through the DUF1669 domain to mediate canonical Wnt signalling.

**Methods:** Co-immunoprecipitation was used to investigate possible changes to PAWS1 interactors caused by the mutations. We also compared the stability of wild-type and mutant PAWS1 in cycloheximide-treated cells. Effects on Wnt signalling were determined using the TOPflash luciferase reporter assay in U2OS cells expressing PAWS1 mutant proteins. The ability of PAWS1 to induce axis duplication in *Xenopus* embryos was also tested. Finally, we knocked-in the A34E mutation at the native gene locus and measured Wnt-induced AXIN2 gene expression by RT-qPCR.

**Results:** We show that these PAWS1 ^A34E^ and PAWS1 ^R52P^ mutants fail to interact with CK1α but, like the wild-type protein, do interact with CD2AP and SMAD1. Like cells carrying a PAWS1 ^F296A^ mutation, which also abolishes CK1α binding, cells carrying the A34E and R52P mutants respond poorly to Wnt signalling to an extent resembling that observed in *FAM83G* gene knockout cells. Consistent with this observation, these mutants, in contrast to the wild-type protein, fail to induce axis duplication in *Xenopus* embryos. We also found that the A34E and R52P mutant proteins are less abundant than the native protein and appear to be less stable, both when overexpressed in *FAM83G*-knockout cells and when knocked-in at the native *FAM83G* locus. Ala ^34^ of PAWS1 is conserved in all FAM83 proteins and mutating the equivalent residue in FAM83H (A31E) also abolishes interaction with CK1 isoforms.

**Conclusions:** We propose that mutations in PAWS1 cause PPK pathogenesis through disruption of the CK1α interaction and attenuation of Wnt signalling.

## Introduction

FAM83G (also known as PAWS1; Protein Associated With SMAD 1) belongs to the FAM83 family of poorly characterised proteins with which it shares the conserved *DUF1669* ( Domain of Unknown Function) at the N-terminus. The primary sequences of FAM83 proteins reveal little about their biochemical functions, and although the DUF1669 domains of all eight FAM83 members (FAM83A-H) contain pseudo-catalytic phospholipase D-like ‘HKD’ motifs, no PLD activity *in vitro* has been reported to date ^1– 3^.

The first clue to possible physiological functions of PAWS1 came in 2013 from a ‘woolly’ mouse phenotype, in which a large deletion of the *FAM83G* gene (probably resulting in a severely truncated protein) was linked to a rough and matted appearance of the coat ^4^. No further studies analysing biochemical or other possible phenotypic abnormalities in these mice have been reported. Another study reported a single homozygous missense mutation in the *FAM83G* gene (c.155C>G), which results in the substitution of a conserved arginine into proline (p.R52P) in the PAWS1 protein. This is the causative genetic defect for hereditary footpad hyperkeratosis (HFH), an autosomal recessive disease affecting several dog breeds, in which gradual thickening of the footpad epidermis leads to the development of painful cracks and fissures. Dogs with HFH also exhibit a softer, duller coat appearance ^5, 6^, reminiscent of the “woolly” mouse phenotypes. The HFH phenotypes also occur in human patients and are broadly described as palmoplantar keratodermas (PPK), which represent a group of skin conditions characterised by thickening of the skin on the palms of the hands and soles of the feet. PPKs often arise from mutations in genes encoding for the keratin cytoskeleton or cell junctions, although there are many cases for which the molecular basis has yet to be established ^7, 8^. Recently, a study reported a single homozygous missense mutation in the *FAM83G* gene (c.101C>A), which results in a substitution of a conserved alanine into glutamate (p.A34E) on the PAWS1 protein. The two human patients were siblings and both presented with palmoplantar keratoderma and thick, exuberant scalp hair ^9^. Both A34E (human) and R52P (dog) mutations in PAWS1 lie within the conserved DUF1669 domain. The high degree of similarity between the phenotypes seen in mice, dogs, and humans provides compelling genetic evidence for the involvement of PAWS1 in skin and hair homeostasis.

In the last few years, we have made progress in understanding the biochemical functions and regulation of PAWS1 and other FAM83 proteins. We discovered that FAM83 proteins, through their DUF1669 domains, interact with distinct sets of CK1α, δ, and ε isoforms to direct them to distinct subcellular locations, thereby, perhaps, regulating the diverse roles of CK1 isoforms ^10^. In particular, we found that PAWS1 interacts with CK1α and that this interaction is essential to promote canonical Wnt signalling in human cell lines and *Xenopus* embryos by accentuating the nuclear accumulation of β-catenin ^1^. In the nucleus, β-catenin forms a complex with TCF/LEF transcription factors to activate Wnt-dependent target gene expression ^11^. Because the Wnt signalling pathway plays crucial roles at several stages of epithelial and hair development (reviewed in 12, 13), we asked whether the pathogenic palmoplantar keratoderma effects of the PAWS1 mutations might be due to dysregulation of Wnt signalling or, alternatively, to other activities of the PAWS1 protein. These other activities include the ability of PAWS1 to associate with the transcription factor SMAD1 to control a subset of non-canonical bone morphogenetic protein (BMP)-induced gene transcription, as well as its ability to interact with the SH3 adaptor CD2AP to regulate actin cytoskeleton remodelling ^14, 15^.

## Methods

### Plasmids and antibodies

All constructs were sequence-verified by the DNA Sequencing Service, University of Dundee. For transient expression or production of retroviral vectors, the following were cloned into pBabe puro plasmids (Cell Biolabs, RTV-001-PURO), with slight modifications introduced at the cloning sites: GFP (DU32961), PAWS1 (DU33460) PAWS1 ^A34E^ (DU28382), PAWS1 ^R52P^ (DU24544), PAWS1 ^F296A^ (DU28044), PAWS1 ^S614A^ (DU33463), PAWS1-GFP (DU29088), PAWS1 ^A34E^-GFP (DU29572), PAWS1 ^R52P^-GFP (DU28090), and PAWS1 ^F296A^-GFP (DU29571). The following were cloned into pcDNA5-FRT/TO-FLAG plasmids (Thermo Fisher Scientific, V652020): FLAG-FAM83H ^WT^ (DU28811), FLAG-FAM83H ^A31E^ (DU29553), FLAG empty (DU41457). HA-SMAD1 (DU19263) was cloned into pCMV5 (DU2865), and CD2AP-FLAG (DU24770) into pcDNA5 (Thermo Fisher Scientific, V652020). M50 Super 8x TOPFlash was a gift from Randall Moon (Addgene plasmid # 12456; RRID:Addgene_12456). Details of plasmids used for CRISPR/Cas9 genome editing are provided as referred to in the text. For transcription of mRNA used in *Xenopus* experiments, sequences were cloned into pCS2: PAWS1-HA (DU64256), PAWS1 ^A34E^-HA (DU64302), and PAWS1 ^R52P^-HA (DU64303). These constructs are available to request from the MRC-PPU reagents webpage and the unique identifier (DU) numbers indicated above provide direct links to the cloning strategies and sequence details.

Antibodies include PAWS1 (S876C, sheep polyclonal, 1:1000; or Abcam, rabbit polyclonal, 1:1000, ab121750), SMAD1 (S618C, sheep polyclonal, 1:1000), CK1α (SA527, sheep polyclonal, 1:1000; or Bethyl Laboratories #A301-991A-M, rabbit polyclonal, 1:1000), GFP (MBL #598, rabbit polyclonal, 1:1000), GAPDH (CST #5174, rabbit polyclonal, 1:5000), α-tubulin (Thermo Fisher Scientific #MA1-80189, rat monoclonal, 1:5000), c-Myc (CST #5605, rabbit monoclonal, 1:1000), FAM83H (SA273, sheep polyclonal, 1:1000), monoclonal mouse anti-FLAG M2-HRP (Sigma, A8592, 1:5000), HA-tag (Sigma, H9658, mouse monoclonal, 1:10000), CK1ε (Sigma, HPA026288, rabbit polyclonal, 1:1000), CK1δ (SA609, sheep polyclonal, 1:1000) and β-actin (CST, #4967S, 1:1000). Unless stated otherwise, antibodies were diluted in TBS buffer (50 mM Tris–HCl, pH 7.5, 150 mM NaCl) containing 5% non-fat dry milk.

Secondary antibodies used for immunoblotting are as follows: IRDye 800CW Donkey anti-Goat (LI-COR, 926-32214, 1:5000), IRDye 800CW Donkey anti-Mouse IgG (LI-COR, 926-32212, 1:5000), IRDye 800CW Donkey anti-Rabbit IgG (LI-COR, 926-32213, 1:5000), IRDye 800CW Goat anti-Rat IgG (LI-COR, 926-32219, 1:5000), IRDye 680LT Donkey anti-Mouse IgG (LI-COR, 926-68022, 1:5000), IRDye 680LT Donkey anti-Rabbit IgG (LI-COR, 926-68023, 1:5000), StarBright Blue 700 Goat Anti-Rabbit IgG (Bio-Rad, 12004161, 1:5000), StarBright Blue 700 Goat Anti-Mouse IgG (Bio-Rad, 12004158, 1:5000), Goat anti-Rabbit IgG HRP (CST #7074, 1:5000), IRDye 800CW Goat anti-Mouse (LI-COR, 926-32210, 1:10,000), IRDye 680LT Goat anti-Rabbit (LI-COR, 926-68021, 1:10,000), and Rabbit anti-Sheep IgG HRP (Thermo Fisher Scientific, 31480, 1:5000).

### Cell culture and transfections

U2OS osteosarcoma (ATCC, HTB-96), HEK293 human embryonic kidney (ATCC, CRL-1573), HaCaT human keratinocytes (from Joan Massague’s lab at Memorial Sloan Kettering Cancer Center, not commercially obtained but can be provided on request) ^16^, mouse fibroblast L-cells that stably overexpress Wnt3A (ATCC, CRL-2647) or L cells (ATCC, CRL-2648) were grown in Dulbecco’s Modified Eagle’s Medium (DMEM; Invitrogen, 11960-085) supplemented with 10% (v/v) FBS (Sigma, F7524), 2 mM L-glutamine (Invitrogen, 25030024), 100 units/ml Penicillin and 100 μg/ml Streptomycin (Invitrogen, 15140122). Cells were grown at 37°C in a humidified incubator at 5% CO _2_. Lipofectamine 2000 (Thermo Fisher Scientific, 11668019) was used for transient transfections with plasmids according to the manufacturer’s recommendation, using a ratio of 1 µg plasmid to 2 µl reagent.

### Preparation of protein extracts

Cells were placed on ice and collected by scraping in ice-cold PBS. The resulting cell pellet was washed with PBS, and either lysed immediately as described below, or stored at -20°C until analysis. Cell pellets were thawed on ice and resuspended in a suitable volume of lysis buffer (50 mM Tris–HCl pH 7.5, 1 mM EGTA, 1 mM EDTA, 1 mM activated Na _3_VO _4_, 10 mM Na β-glycerophosphate, 50 mM NaF, 5 mM Na Pyrophosphate, 270 mM sucrose, 1% (v/v) NP-40 substitute (Merck, 492016) and a protease inhibitor cocktail (Merck, 11873580001). After 10 min incubation on ice, lysates were clarified by centrifugation at 13,000 × g for 15 min at 4°C. Supernatant was recovered (soluble cell extract), and protein concentration was determined in a 96-well format using Bradford protein assay reagent (Pierce, 23236). Absorbance at 595 nm was measured using the Epoch microplate spectrophotometer (BioTek).

*Xenopus* extracts were prepared by titrating with 10 μl/embryo of embryo lysis buffer (1% IGEPAL, 150 mM NaCl, 10 mM HEPES pH 7.4, 2 mM EDTA, protease inhibitor cocktail (Pierce, A32965)). Lipids and yolk were removed by extracting the lysate with an equal volume of 1,1,2-Trichloro-1,2,2-trifluoroethane (FREON, Sigma Aldrich, 130400).

### Immunoprecipitation and immunoblotting

0.3 – 1 mg of soluble cell extract protein was incubated with 10 µl of GFP-Trap beads (ChromoTek, gta-10), M2 anti-FLAG Sepharose (Sigma, A2220), or PAWS1 antibody and Protein G-Sepharose (Sigma, P3296) for 1–2 h at 4°C with gentle agitation. Beads were then washed 5 times with lysis buffer. Immunoprecipitated proteins and protein extracts (10–20 µg) were denatured in SDS sample buffer and then separated by SDS-PAGE. Proteins were transferred to 0.2 µM pore size nitrocellulose membrane (GE Healthcare, 10600001). The membrane was blocked with 5% non-fat dry milk in TBS buffer (50 mM Tris–HCl, pH 7.5, 150 mM NaCl) or Odyssey Blocking Buffer in TBS (LI-COR, 927-50000) for 1 h and then with primary antibody diluted in blocking solution containing 0.1% (v/v) Tween-20 overnight at 4°C. Blots were incubated for 1 h at room temperature with the appropriate IRDye (LI-COR), StarBright (Bio-Rad) fluorescently conjugated or HRP conjugated secondary antibodies diluted in blocking solution, and visualised using the Odyssey Imager (LI-COR) or Chemidoc MP system (Bio-Rad, 17001402).

### Generation of PAWS1 and FAM83H knockouts and A34E knock-in cells by CRISPR/Cas9

To generate PAWS1 knockouts, cells were transfected with vectors encoding a pair of guide RNAs (pBabeD-Puro-gRNA1 (GGACCGCTCCATCCCGCAGC) and pX335-CAS9-D10A-gRNA2 (GCTGGGGCCAGTACTCCAGGG), DU52480 and DU52484 respectively) targeting the first coding exon (Exon 2) of *PAWS1*. A similar approach was used to generate FAM83H knockouts, with guide RNAs (DU52010 and DU52026) targeting the first coding exon (Exon 2) of *FAM83H* ^10^ *.* For the knock-in of the PAWS1 A34E point mutation, a single guide RNA targeting Exon 2 was used (pX459-Puro-CAS9-sgRNA (GCTACAGCGAGGAGCAGCGGC), DU60688). The plasmid donor (DU60974) contains a GFP sequence (allowing single cell FACS selection for donor integration), and an internal ribosome entry site (IRES) upstream of the PAWS1 transcription start site. In addition to the point mutation for A34E (c.101C>A), silent base substitutions were made to prevent further recognition by the gRNA, and to introduce a *XhoI* restriction site to allow screening. 24 h post-transfection with the indicated plasmids, cells were cultured with 2 µg/ml puromycin (Sigma, P9620). Surviving cells were sorted on an Influx cell sorter (Becton Dickinson) equipped with a 488nm laser, using PBS as sheath at a pressure of 6 psi through a 140 µm nozzle. Live cells were distinguished from debris based on forward scatter-height (FSC-H) v side scatter-height (SSC-H) measurements, and single cells distinguished from doublets based on FSC-Area (A) v FSC-Width (W). Where GFP positive cells were collected, the autofluorescence of non-fluorescent control cells was assessed by measuring fluorescence emission at 530±40nm and 580±30nm. GFP positive cells were identified in subsequent samples as exhibiting 530±40nm fluorescence above that of controls. Viable clones were verified by genomic sequencing and immunoblotting.

For verification by DNA sequencing, the region surrounding the gRNA target sites were amplified by PCR with KOD Hot Start Polymerase (Merck, 71086) according to manufacturer’s instructions with the following primer pairs for PAWS1 KO and PAWS1 ^A34E^ knock-in respectively: F: TCTTTCCCGCAGATTGCTCATGG, R: TTCTTCTGGGGAACCAGAAACACC; F: TGGACGACAACCATGTGAACTGG, R: CGCACCACCTCTTTGATGTGG. Cycling conditions: 98°C 2 min, (98°C 10 sec, 60°C 15 sec, 70°C 30 sec) ×40 cycles, 70°C 5 min. Amplification was performed using a DNA Engine thermal cycler (BioRad, ALS-1296G). PCR products were ligated into a sequencing vector using the StrataClone Blunt PCR Cloning Kit (Agilent, 240207) according to manufacturer’s instructions. Resulting plasmid clones were sequenced by the DNA Sequencing Service, University of Dundee using the M13 Forward primer (GTAAAACGACGGCCAGTG).

### Retroviral transduction of cells for the stable expression of target proteins

Retroviral pBabe-puromycin vectors encoding GFP or the desired target protein (6 μg) were co-transfected with pCMV5-gag-pol (3.2 μg, Cell Biolabs, RV-111) and pCMV5-VSV-G (2.8 μg, Cell Biolabs, RV-110) into a 10 cm-diameter dish of ~70% confluent HEK293-FT cells. Briefly, plasmids were added to 1 ml Opti-MEM medium (Thermo Fisher Scientific, 31985062) to which 24 μl of 1 mg/ml polyethylenimine (PEI; diluted in 25 mM HEPES pH 7.5) was added. Following a brief vortex mix and incubation at room temperature for 20 min, the transfection mix was added dropwise to the HEK293-FT cells. 16 h post-transfection, fresh medium was added to the cells. 24 h later, the retroviral medium was collected and passed through 0.45 μm filters. Target PAWS1-KO HaCaT or U2OS cells (~60% confluent) were infected with the optimised titre of the retroviral medium diluted in fresh medium (typically 1:5 – 1:10) containing 8 µg/ml polybrene (Sigma, H9268) for 24 h. The retroviral infection medium was then replaced with fresh medium, and 24 h later, the medium was again replaced with fresh medium containing 2 µg/ml puromycin (Sigma, P9620) for selection of cells which had integrated the rescue constructs.

### Mass spectrometry

10.5 mg of protein in soluble cell extract from HaCaT cells was pre-cleared by incubation with Protein G-Sepharose for 30 min at 4°C, then incubated with GFP-Trap beads (ChromoTek, gta-10) for 4 h at 4°C. Beads were washed 5 times with lysis buffer, then denatured in LDS sample buffer (Thermo Fisher Scientific, NP0007) supplemented with 2% β-mercaptoethanol. Samples were filtered through a Spin-X centrifuge tube filters (Sigma, CLS8161), resolved by 4–12% gradient SDS–PAGE (Thermo Fisher Scientific, NP0323), and stained with colloidal Coomassie blue. Gels were destained in Milli-Q H _2_O until background staining was minimal. Sections of the gel were excised, trypsin digested, and peptides prepared for analysis.

Mass spectrometric analysis was performed by LC-MS-MS (Liquid Chromatography-tandem Mass Spectrometry) on a Linear ion trap-orbitrap hybrid mass spectrometer (Orbitrap-VelosPro, Thermo) coupled to a U3000 RSLC HPLC (Rapid Separation/High-Performance Liquid Chromatography; Thermo). Peptides were trapped on a nanoViper Trap column, 2cm × 100µm C18 5µm 100Å (Thermo, 164564) then separated on a 50cm Thermo EasySpray column (ES803) equilibrated with a flow of 300 nl/min of 3% Solvent B. [Solvent A 0.1% formic acid; Solvent B 80% acetonitrile, 0.08% formic acid]. The elution gradient was as follows, Time(min):Solvent B(%); 0:3, 5:5, 45:35, 47:95, 52:95, 52.5:3, 65:3. The instrument was operated with the “lock mass” option to improve the mass accuracy of precursor ions and data were acquired in the data-dependent mode, automatically switching between MS and MS-MS acquisition. Full scan spectra (m/z 400-1600) were acquired in the orbitrap with resolution R = 60,000 at m/z 400 (after accumulation to an FTMS (Fourier Transform Mass Spectrometry) Full AGC (Automatic Gain Control) Target; 1,000,000; FTMS MSn AGC Target; 50,000). The 20 most intense ions, above a specified minimum signal threshold (2,000), based upon a low resolution (R = 15,000) preview of the survey scan, were fragmented by collision induced dissociation and recorded in the linear ion trap, (Full AGC Target; 30,000. MSn AGC Target; 5,000).

Data files were analysed by Proteome Discoverer 2.0 (Thermo), using Mascot 2.4.1, and searching against SwissProt database allowing for the following peptide modifications, Carbamidomethyl (C) – fixed modification, and Oxidation (M), Dioxidation (M) as variable modifications. Error tolerances were 10ppm for MS1 and 0.6 Da for MS2. Scaffold 4 was also used to examine the Mascot result files.

### Dual luciferase reporter assays

HEK293 cells (4 × 10 ^4^/cm ^2^) were seeded in 12-well plates. 24 h later, 200 ng of SuperTOPFlash, 20 ng of Renilla luciferase (Promega, E2261), and 100 ng of GFP or PAWS1-GFP plasmids were co-transfected as described above. After 24 h, cells were treated with L-Wnt3A or L-conditioned medium for 6 h. Cells were washed with PBS and lysed in Passive Lysis Buffer (Promega, E194A). Firefly and Renilla luciferase activities were measured as described previously ^17^. Briefly, extracts were mixed 1:1 with 2x Luciferase Buffer (50 mM Tris/phosphate (pH 7.8), 16 mM MgCl _2_, 2 mM DTT (dithiothreitol), 30% (v/v) glycerol, 1 mM ATP, 1% BSA, 0.25 mM luciferin and 8 μM sodium pyrophosphate) and light emission was measured using a Envision 2104 plate reader (PerkinElmer). An equivalent volume of 3x Renilla Assay Buffer (45 mM Na _2_EDTA, 30 mM sodium pyrophosphate, 1.425 M NaCl, 60 µM PTC124, 10 µM h-Coelenterazine) was then added, and Renilla luciferase emission was measured. The firefly luciferase counts were normalised to Renilla for each sample.

### Quantitative PCR and primers

Total RNA was isolated from cells using the RNeasy Micro kit (Qiagen, 74004). Reverse transcription was performed using 1 μg of isolated RNA and the iScript cDNA synthesis kit (Bio-Rad, 170–8891) according to the manufacturer’s protocol. Quantitative PCR was performed in 10 μl reaction volumes with three or four technical replicates. Each reaction included 2 μM forward and reverse primers, PowerUp SYBR Green Master Mix (Thermo Fisher Scientific, A25742), and cDNA equivalent to 10 ng of RNA, and monitored in a CFX384 real-time PCR detection system (Bio-Rad, 1855485). Cycling conditions: 50°C 2 min, 95°C 2 min, (95°C 10 sec, 60°C 30 sec) ×45 cycles. Ct values were determined by the CFX Manager 3.1 software (Bio-Rad, 1845000), and relative gene expression was determined using the delta-delta Ct method.

Primers: GAPDH forward (TGCACCACCAACTGCTTAGC), GAPDH reverse (GGCATGGACTGTGGTCATGAG), AXIN2 forward (TACACTCCTTATTGGGCGATCA), AXIN2 reverse (TTGGCTACTCGTAAAGTTTTGGT), PAWS1 forward (CACAGAAGGTGATAGCTGTG), PAWS1 reverse (ACTTGACGTTACTCTCATCCA). All graphs shown are the result of at least three biological replicates.

### Cycloheximide block

U2OS cells (2 × 10 ^4^/cm ^2^) were seeded in 6-well plates. 24 h later, cells were transfected with 750 ng pBabe PAWS1 plasmids per well. The following day, cells were treated with 100 µg/ml cycloheximide (Sigma, C7698) and/or 5 µM bortezomib (Sigma, 5043140001) for the indicated times prior to collection.

### *Xenopus laevis* assay methodology

All *Xenopus laevis* work, including general housing and husbandry, was undertaken in accordance with The Crick Use of animals in research policy, the Animals (Scientific Procedures) Act 1986 (ASPA) implemented by the Home Office in the UK and the Animal Welfare Act 2006. Consideration was given to the ‘3Rs’ in experimental design. *Xenopus laevis* embryos were obtained by *in vitro* fertilisation and staged according to Nieuwkoop and Faber (1975). Embryos were maintained in Normal Amphibian Medium (NAM) at 21°C, 18°C or 14°C until the 4-cell stage was reached. Embryos were then injected into a single ventral blastomere with 500 pg of the indicated capped RNA, synthesised using SP6 mMessage mMachine kit (Invitrogen, AM1340), in a total volume of 5 nl. Embryos were then allowed to develop to approximately stage 34–35 at 21°C, before being fixed in 4% paraformaldehyde (PFA). Embryos were then counted and scored for the secondary axis phenotype: secondary axis complete with 2 × cement gland = complete secondary axis; secondary axis apparent but 1 × cement gland = partial secondary axis; enlarged cement gland/rostral structures = dorsalised; comparable to WT = WT. As phenotypes were distinct, no blinding/randomisation was undertaken. For each experiment, approximately 35–40 embryos were injected to ensure sufficient statistical power and a similar number of uninjected embryos were kept under the same conditions as controls. The experiment was repeated three times, twice on the same day (morning and afternoon) using eggs from two different females and testes from the same male, the third experiment was undertaken on a separate day using eggs from a third female and testes from a second male.

For western blotting, embryos were obtained as described above and injected with 500 pg of the indicated capped RNA into the animal hemisphere at the one-cell stage. Embryos were allowed to develop to stage 10 at 18°C before being snap frozen on dry ice and stored at -20°C for later protein extraction.

### Statistical analysis

Graphing and statistical tests were performed using Prism 7 software (GraphPad). Unless otherwise noted, data are presented as the mean ± standard deviation of at least three biological replicates. Specific tests used are described in the respective figure legends. Significance levels are as follows: *P<0.05, **P<0.005, ***P<0.001. See underlying data for data underlying all presented figures ^18^.

## Results

### Palmoplantar keratoderma (PPK)-associated PAWS1-A34E and R52P mutants interfere with CK1α binding

**Figure 1.**
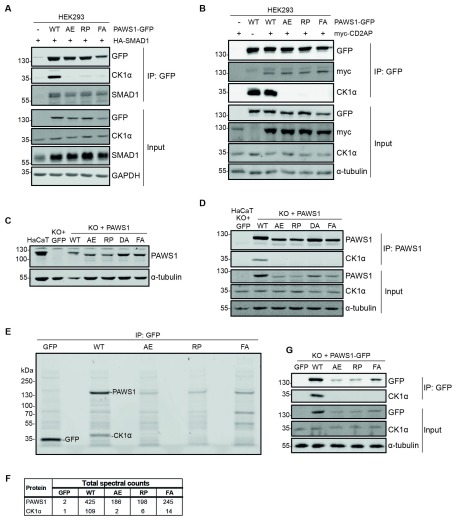
Pathogenic PPK point-mutations in PAWS1 disrupt its interaction with CK1α. **A**, **B**: HEK293 cells transiently expressing PAWS1-GFP and HA-SMAD1 ( **A**) or myc-CD2AP ( **B**) were subject to GFP immunoprecipitation (IP) and immunoblotting (IB) for the indicated proteins. **C**: Using retroviral transduction, PAWS1 or the indicated mutants were stably re-expressed in HaCaT PAWS1 ^−/−^ (KO) cells; GFP control, wildtype (WT), A34E (AE), R52P (RP), D262A (DA), F296A (FA). Cell extracts were analysed by IB. **D**: Immunoprecipitation of PAWS1 was performed from HaCaT cells described in ( **C**). **E**– **G**: PAWS1-GFP or the indicated mutants were stably expressed in PAWS1-KO HaCaT cells as described in ( **C**), and immunoprecipitated with GFP-Trap beads. GFP IP samples were separated by SDS-PAGE and Coomassie stained ( **E**). Each lane was cut into 6 pieces and subsequently processed for protein identification by mass spectrometry. Table showing total spectral counts for PAWS1 and CK1α ( **F**). Input and IP samples were analysed by IB with the indicated antibodies ( **G**).

PAWS1 interacts with CK1α ^1, 10^, SMAD1 ^15^, and CD2AP ^14^. We first asked whether the two PPK-associated mutants A34E and R52P affect the ability of PAWS1 to interact with these three proteins. To this end, we co-expressed PAWS1-GFP in HEK293 cells with either HA-SMAD1 or myc-CD2AP. Immunoprecipitation (IP) experiments confirmed that PAWS1-GFP co-precipitates with HA-SMAD1 ( Figure 1A) and myc-CD2AP ( Figure 1B), as well as with endogenous CK1α. Under these conditions, both PAWS1 ^A34E^ and PAWS1 ^R52P^ also co-precipitate with HA-SMAD1 ( Figure 1A) and myc-CD2AP ( Figure 1B) but did not immunoprecipitate with endogenous CK1α ( Figure 1A&B). As a control, we made use of the PAWS1 ^F296A^ mutant, which lies within the DUF1669 domain of PAWS1 and is unable to interact with CK1α ^1^. Like the A34E and R52P mutants, PAWS1 ^F296A^ did not interact with CK1α ( Figure 1A&B), but did co-precipitate with HA-SMAD1 ( Figure 1A) and myc-CD2AP ( Figure 1B).

PPK phenotypes are associated with abnormal epidermis and often result from epidermal hyperplasia ^7, 19, 20^. To investigate the impact of the PPK mutants in a physiologically relevant cell line model, we used CRISPR/Cas9 genome editing to generate PAWS1-knockout (KO) HaCaT cells, which are a spontaneously transformed human keratinocyte cell line ( Figure 1C & Figure S1 (extended data ^18^)). These cells were then stably restored with near-endogenous levels of wild-type PAWS1, or with equivalent levels of the two pathogenic mutants (A34E & R52P) and two CK1-interaction deficient mutants (D262A & F296A) ^1^ or the GFP control ( Figure 1C). As we had previously reported with U2OS cells ^1^, PAWS1 ^WT^ displayed predominantly diffused cytoplasmic localisation in HaCaT cells, and no obvious differences in localisation patterns were observed with PAWS1 ^A34E^ or PAWS1 ^R52P^ (Figure S2 (extended data ^18^)). Endogenous CK1α was detected in PAWS1 IPs from cells rescued with PAWS1 ^WT^ but not from those rescued with the pathogenic mutants or with the CK1-interaction deficient mutants ( Figure 1D).

To ask whether the pathogenic PPK PAWS1 mutants affect PAWS1 function through additional changes to interacting partners, we undertook an unbiased proteomic approach to identify interactors of these mutants. To this end, PAWS1-KO HaCaT cells were rescued with GFP control, PAWS1-GFP, PAWS1 ^A34E^-GFP, PAWS1 ^R52P^-GFP or PAWS1 ^F296A^-GFP, and anti-GFP IPs were subjected to proteomic analyses. A Coomassie-stained gel revealed that the disappearance of a band at ~41 kDa, the predicted size of endogenous CK1α, from IPs of PAWS1 ^A34E^, PAWS1 ^R52P^ and PAWS1 ^F296A^ was the only striking difference from the wild-type control ( Figure 1E). Proteomic analysis of interacting proteins from each IP confirmed that the only difference between PAWS1 ^WT^ and the three mutants was in the abundance of CK1α ( Figure 1F), suggesting that non-interaction of the mutants with CK1α is likely to be a key factor in pathogenesis of PPK. This was further verified by immunoblotting, which showed that PAWS1 ^WT^ interacts with endogenous CK1α while the mutants do not ( Figure 1G).

Intriguingly, the mutant proteins had a lower apparent molecular weight on SDS-PAGE compared with the WT protein ( Figure 1C, D, E & G). As PAWS1 is a known substrate of CK1α ^1^, we investigated if this mobility shift reflected a change in the phosphorylation status. Indeed, PAWS1 WT was observed as a single, faster migrating band following treatment with lambda protein phosphatase *in vitro* (Figure S3 (extended data ^18^)). PAWS1 ^A34E^ and PAWS1 ^R52P^ also appear to be phosphorylated in cells, albeit to a lesser extent. Meanwhile, the mobility shift of PAWS1 ^S614A^ (which interacts with, but is not phosphorylated by CK1α ^1^) is indistinguishable from PAWS1 ^WT^. Thus, PAWS1 phosphorylation by other kinases can occur in both CK1α-dependent and independent manner.

Furthermore, when PAWS1-KO cells were rescued with PAWS1 ^WT^ or with the PPK pathogenic mutants, we consistently observed a lower abundance of PAWS1 ^A34E^ and PAWS1 ^R52P^ proteins compared with PAWS1 ^WT^ ( Figure 1C, D, E & G). This suggests that the A34E and R52P mutations might affect the stability of PAWS1 protein.

### PPK PAWS1 mutants exhibit reduced protein stability

**Figure 2.**
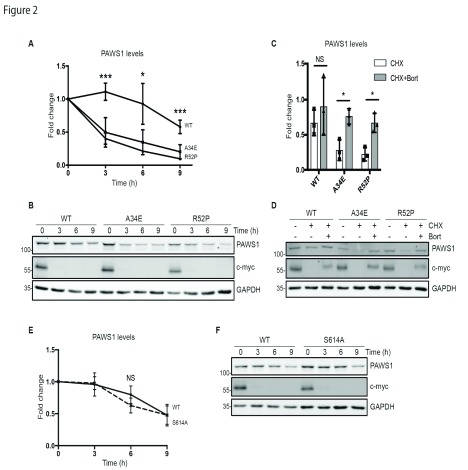
PAWS1 ^A34E^ and PAWS1 ^R52P^ proteins have a shorter half-life in cells. **A**, **B**: U2OS PAWS1-KO cells transiently expressing PAWS1 ^WT^, PAWS1 ^A34E^, or PAWS1 ^R52P^ were treated with 100 µg/ml cycloheximide for the indicated times prior to sample collection. PAWS1 band intensities were measured, normalised to GAPDH loading control, and represented relative to the respective 0 h samples (n=3, error bars represent ± SD). Representative blots are shown in ( **B**). Two-way ANOVA. **C**, **D:** As in ( **A**, **B**), but indicated cells were treated with cycloheximide in the presence or absence of bortezomib (5 µM) for 6 h. Fold changes are shown relative to the respective untreated samples. Representative blots are shown in ( **D**). Multiple t-test. **E**, **F**: Cycloheximide chase performed as described in ( **A**) with PAWS1 ^WT^ and PAWS1 ^S614A^. Representative blots are shown in ( **F**). Two-way ANOVA.

To ask whether the pathogenic mutations of PAWS1 affect its stability, we first transiently transfected PAWS1 ^WT^, PAWS1 ^A34E^, and PAWS1 ^R52P^ into PAWS1-KO U2OS osteosarcoma cells to achieve comparable starting levels of the respective proteins. The stability of the proteins was tested over 9 h following inhibition of protein synthesis with cycloheximide ( Figure 2A&B). We found that following cycloheximide treatment, PAWS1 ^A34E^ and PAWS1 ^R52P^ protein levels declined more rapidly (t _1/2_ = 3 h) than PAWS1 ^WT^ (t _1/2_ > 9 h). After 9 h of cycloheximide treatment, PAWS1 ^A34E^ and PAWS1 ^R52P^ protein levels were reduced to 10–20% of the levels of their untreated controls (0 h), while about 60% of PAWS1 ^WT^ remained ( Figure 2A&B). As a control, c-myc protein levels were undetectable within 3 h of cycloheximide treatment ( Figure 2B). The reductions in PAWS1 ^A34E^, and PAWS1 ^R52P^ protein levels, as well as that of c-myc, was rescued by co-treatment with the proteasome inhibitor bortezomib ( Figure 2C&D).

One possible explanation for the decreased stability of the PAWS1 ^A34E^ and PAWS1 ^R52P^ proteins is that inability to interact with CK1α prevents their phosphorylation by CK1α. However, PAWS1 ^S614A^ is not phosphorylated by CK1α ^1^, and its stability is unaffected in the cycloheximide assay ( Figure 2E, F), arguing that PAWS1 phosphorylation by CK1α does not regulate its stability.

### Canonical Wnt signalling is impaired by PPK PAWS1 mutations

**Figure 3.**
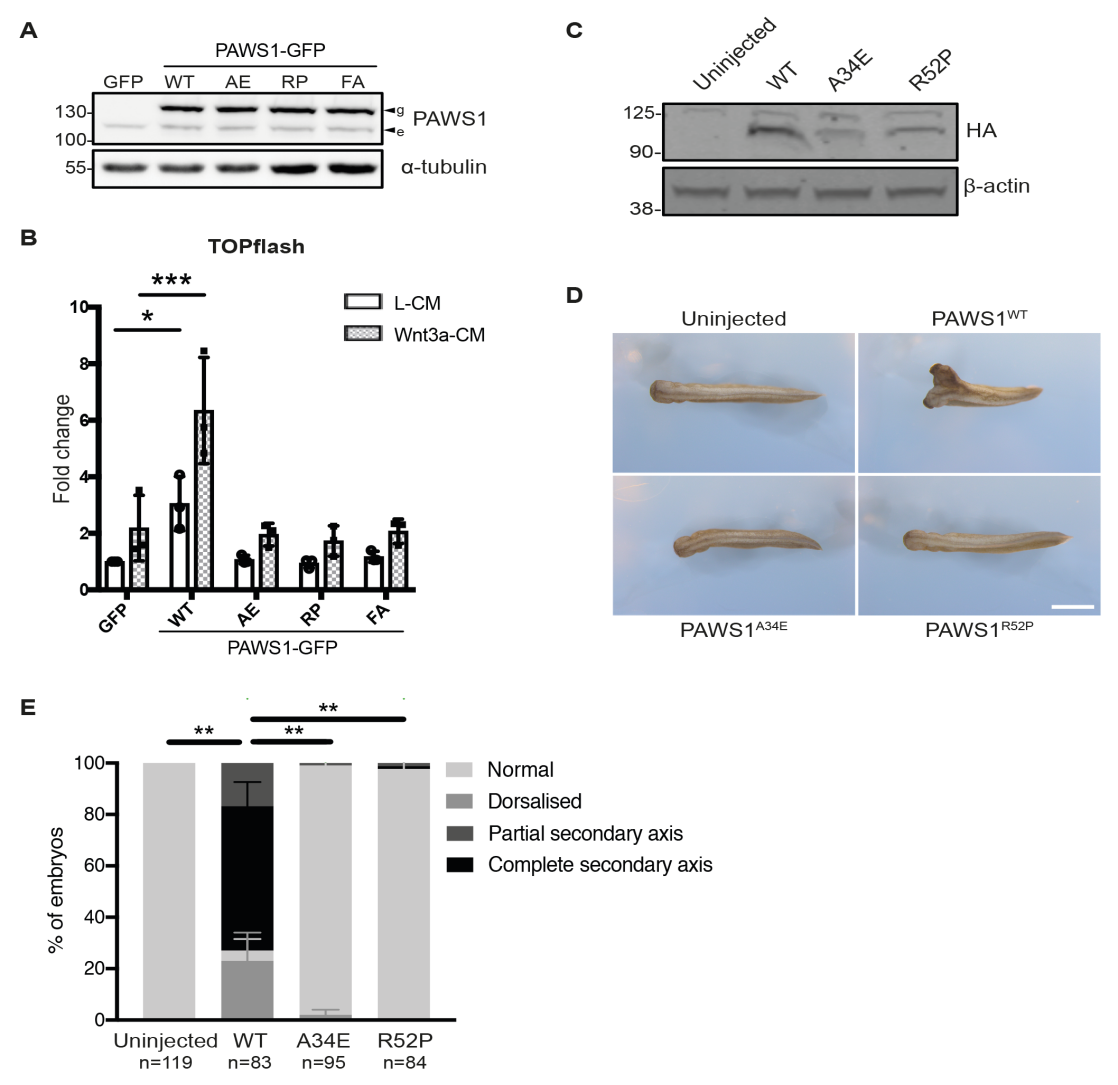
Pathogenic PPK PAWS1 point mutants impact canonical Wnt signalling. **A**, **B**: U2OS cells were transfected with PAWS1-GFP or the indicated mutants of PAWS1 or GFP alone. Levels of endogenous (e) or GFP-tagged (g) PAWS1 in protein extracts were analysed by immunoblotting ( **A**). TOPflash luciferase activity was measured after treatment with either control conditioned medium (L-CM) or Wnt3A conditioned medium (Wnt3a-CM) for 6 h ( **B**). Data are normalised to Renilla luciferase as internal control. Values shown relative to L-CM treated GFP control (n=3). Two-way ANOVA. **C**– **E**: 500 pg hPAWS1 mRNA was injected into *Xenopus* embryos at the four-cell stage. Protein levels were analysed by IB ( **C**). Representative images showing complete axis duplication and normal phenotypes at tadpole stage in injected embryos; scale bar = 1 mm ( **D**). % of embryos showing axis duplication phenotypes were quantified from three independent experiments. Error bars show SD; Two-way ANOVA ( **E**).

The PAWS1-CK1α complex is an important mediator of the Wnt signalling pathway, so we sought to determine if canonical Wnt signalling is affected by the PAWS1 ^A34E^ and PAWS1 ^R52P^ mutants. Because HaCaT cells did not respond to stimulation with Wnt3a (Figure S4 (extended data ^18^)), we turned to the U2OS cells in which we have previously studied canonical Wnt signalling ^1^. We co-expressed PAWS1 ^WT^, PAWS1 ^A34E^, PAWS1 ^R52P^, or PAWS1 ^F296A^ with a TOPflash Wnt/β-catenin luciferase reporter in U2OS cells ^1^, and measured luciferase reporter activity following stimulation with control- or Wnt3A-conditioned medium ( Figure 3A&B). Consistent with our previous report ^1^, overexpression of PAWS1 ^WT^ increased both basal and Wnt3A-stimulated reporter activity compared with GFP and PAWS1 ^F296A^ controls ( Figure 3A&B). Under these conditions, overexpression of the PAWS1 ^A34E^ and PAWS1 ^R52P^ mutants, at similar levels to that of PAWS1 ^WT^, did not enhance either basal luciferase reporter activity or that induced by Wnt3A ( Figure 3A, B), suggesting that these mutants are unable to mediate Wnt signalling in U2OS cells.

Consistent with its Wnt-activating role, we have previously demonstrated that ectopic delivery of PAWS1 ^WT^ mRNA into a single ventral blastomere at the 4-cell stage *Xenopus* embryo results in the formation of a complete secondary axis, resembling that formed in response to ectopic xWnt8. This axis-inducing ability of PAWS1 requires CK1α-binding because the PAWS1 ^D262A^ and PAWS1 ^F296A^ mutants fail to induce axis duplication ^1^. In order to test the axis-inducing ability of PAWS1 ^A34E^ and PAWS1 ^R52P^ mutants, we microinjected PAWS ^WT^ or the mutant mRNAs into *Xenopus* embryos and assessed the formation of a secondary body axis at the tadpole stage. While PAWS ^WT^ induced partial or complete axis duplication in ~80% of embryos, PAWS1 ^A34E^ and PAWS1 ^R52P^ mutants did not ( Figure 3C–E), further confirming the failure of these mutants to activate Wnt signalling. We also observed lower levels of PAWS1 ^A34E^ and PAWS1 ^R52P^ protein relative to PAWS1 ^WT^ in these tadpoles despite the embryos being injected with the same amounts of mRNA ( Figure 3C). Thus, we normalised the amount of PAWS1 ^WT^ and mutant proteins expressed in the embryos by titrating the amount of mRNA injected. Axis duplication phenotypes were still observed in ~50% of embryos injected with half the amount of PAWS1 ^WT^ mRNA, indicating that the lack of phenotype observed with the mutants is unlikely to be explained by reduced protein levels (Figure S5 (extended data ^18^)).

**Figure 4.**
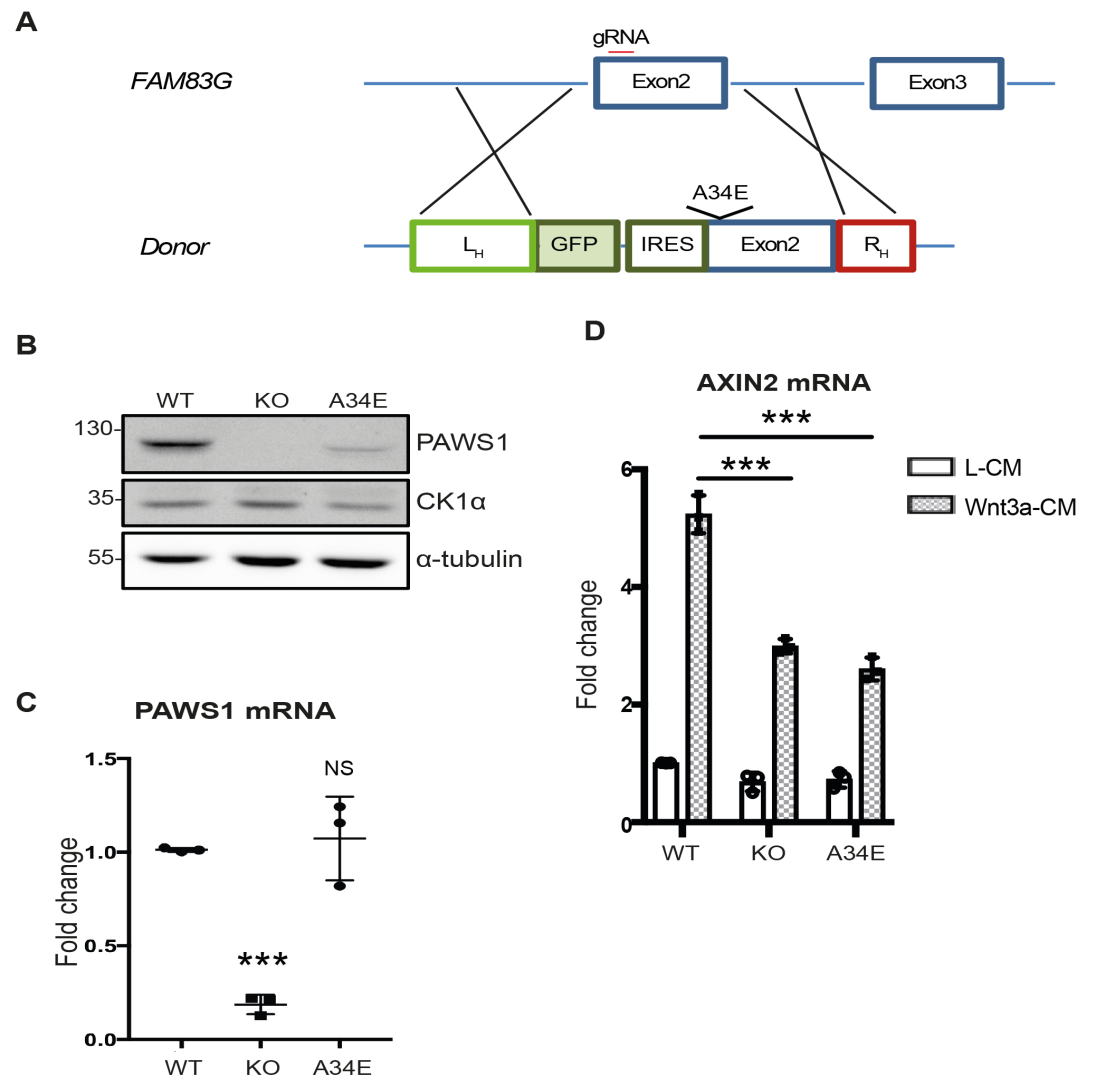
PAWS1 ^A34E^ knock-in reduces protein levels and impairs Wnt signalling. **A**: Schematic overview of CRISPR/Cas9 knock-in strategy. A GFP coding sequence, internal ribosome entry site (IRES), and mutations to the PAWS1 coding sequence in Exon2 were introduced by homology-directed repair with a plasmid donor. Further details are available in the Materials and methods section. L _H_, left homology arm; R _H_, right homology arm. **B**: U2OS, PAWS1-KO, and PAWS1 ^A34E^ KI cell extracts were analysed by IB with the indicated antibodies. **C**: PAWS1 transcript levels relative to GAPDH control in asynchronously growing cultures were assessed by RT-qPCR (n=3), and represented as fold-change relative to U2OS ^WT^. One-way ANOVA. **D**: Cells were treated with L-CM or Wnt3a-CM for 3 h. Expression of AXIN2 was assessed by RT-qPCR relative to GAPDH, and represented as the fold-change over L-CM treated U2OS ^WT^ (n=3). Two-way ANOVA.

To circumvent potential artefacts of the overexpression systems used above, we used CRISPR/Cas9 genome editing to replace the endogenous PAWS1 protein of U2OS cells with PAWS1 ^A34E^. To achieve this, we used a novel donor strategy to knock in a polycistronic cassette consisting of GFP cDNA, an internal ribosome entry site (IRES) element, and PAWS1 ^A34E^ cDNA directly downstream of the native *FAM83G* promoter ( Figure 4A). GFP-positive clones were isolated and homozygous insertion of the PAWS1 ^A34E^ mutation was verified by PCR and genomic sequencing of one of the clones, which was then selected for further investigation (Figure S6 (extended data ^18^)). Consistent with the destabilising effect of the PAWS1 ^A34E^ mutation demonstrated earlier ( Figure 2A–D), PAWS1 protein levels but not mRNA levels in U2OS ^A34E^ cells were substantially lower than in U2OS ^WT^ cells ( Figure 4B&C). We note, however, that this may also be due in part to reduced efficiency of translation initiated by the IRES relative to the wildtype mRNA sequence ^21, 22^. Interestingly, the *PAWS1* mRNA levels in PAWS1-KO U2OS cells, also generated by CRISPR/Cas9 genome editing, were much lower than in U2OS ^WT^ and U2OS ^A34E^ cells ( Figure 4C), probably because of nonsense-mediated decay of the PAWS1-KO transcript caused by a premature stop codon.

We measured Wnt-induced expression of the canonical Wnt target gene *AXIN2* in U2OS ^WT^, U2OS ^KO^, and U2OS ^A34E^ cells. Wnt3a treatment induced a robust 5-fold upregulation of *AXIN2* mRNA in U2OS ^WT^ cells relative to control ( Figure 4D). In contrast, in both U2OS ^KO^ and U2OS ^A34E^ cells, Wnt3A-induced upregulation of *AXIN2* mRNA was significantly reduced compared with U2OS ^WT^ cells; p<0.001 ( Figure 4D).

### Alanine 34 of PAWS1 is conserved in FAM83 proteins and appears functionally analogous in FAM83H

**Figure 5.**
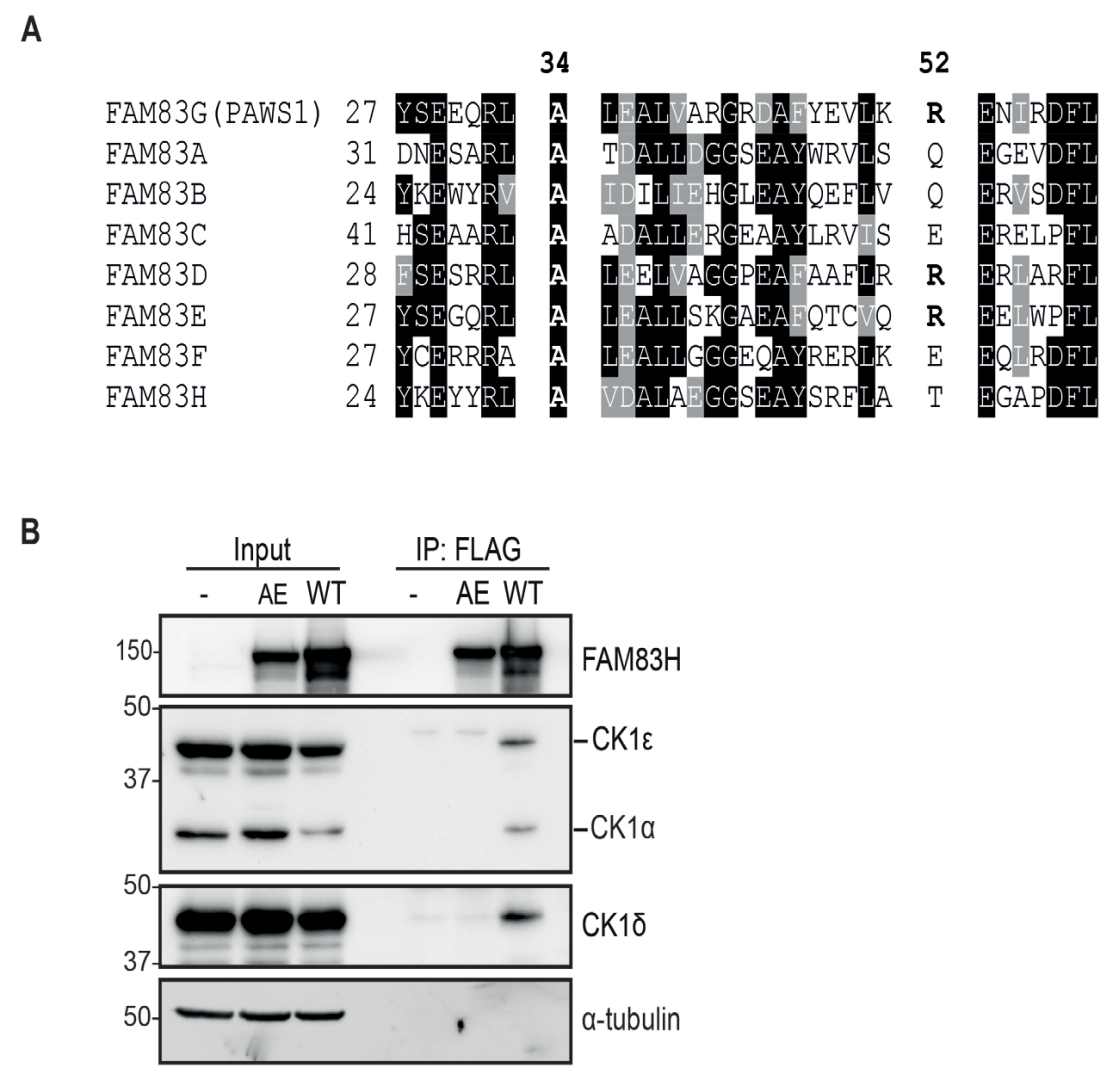
Conservation of the PAWS1 alanine 34 in FAM83A-H. **A**: Multiple sequence alignments were performed using Clustal Omega (EMBL-EBI) and visualised using BoxShade Server (EMBnet). Letters boxed in black indicate identical residues and letters shaded in grey indicate similar residues. Residues that are identical or similar in at least 50% of the FAM83 members are shaded. **B**: FLAG empty vector (-), FLAG-FAM83H ^WT^ (WT) and FLAG-FAM83H ^A31E^ (AE) were transiently expressed in FAM83H-KO U2OS cells. Cells were lysed, FLAG IPs were performed and IPs were analysed by immunoblotting for CK1 isoforms.

Both Ala ^34^ and Arg ^52^ of PAWS1 lie in the DUF1669 domain, which is conserved and located at the N-terminus of FAM83 proteins and is required for binding to CK1 kinases ^10^. Whilst the Arg ^52^ residue of PAWS1 is conserved only in FAM83D and FAM83E, Ala ^34^ is completely conserved across all FAM83 members, and may therefore serve a similar and important function for all FAM83 members ( Figure 5A). With this in mind, we made an analogous mutation on FAM83H (A31E) and introduced FLAG-FAM83H ^WT^ or FLAG-FAM83H ^A31E^ into FAM83H ^KO^ U2OS cells. Interestingly, we observed lower levels of FAM83H ^A31E^ protein than of FAM83H ^WT^ ( Figure 5B), reminiscent of the observation that PAWS1 ^A34E^ is less stable than PAWS1 ^WT^. Consistent with our previous report ^10^, IPs of FAM83H ^WT^ co-precipitated endogenous CK1α, δ, and ε isoforms ( Figure 5B). However, FAM83H ^A31E^ IPs did not co-precipitate CK1α, δ, or ε isoforms ( Figure 5B), suggesting that this residue in FAM83 proteins is necessary for binding to CK1 isoforms.

## Discussion

The almost identical hyperproliferative epidermis and hair phenotypes reported in human PPK patients carrying the homozygous PAWS1 ^A34E^ mutation ^9^ and in HFH dogs carrying the PAWS1 ^R52P^ mutation ^5, 6^ hint at a common mechanism for disease pathogenesis. Our results strongly suggest that this common mechanism involves the inability of PAWS1 ^A34E^ and PAWS1 ^R52P^ to associate with CK1α, which reduces their ability to activate Wnt signalling.

CK1α (and other CK1 isoforms) regulates Wnt signalling both positively and negatively by phosphorylating many components of the pathway. For example, CK1α phosphorylates cytoplasmic β-catenin at Ser45, which allows GSK3β to phosphorylate Thr41, Ser37 and Ser33 and mark it for proteasomal degradation, thus down-regulating Wnt/β-catenin signalling ^23, 24^. In contrast, CK1 kinases can also positively regulate Wnt signalling ^25^. For example, in response to the binding of Wnt ligand to the LRP5/6 receptor, CK1α phosphorylates LRP5/6 and p120-catenin at the plasma membrane, both of which events are needed for full activation of signalling ^26, 27^.

We have previously shown that CK1α can exist in distinct complexes with all FAM83 members in addition to PAWS1 ^10^. Individual FAM83 proteins deliver CK1α or other CK1 isoforms to distinct subcellular compartments, and potentially to specific CK1 substrates, to influence specific cellular processes. For example, FAM83D delivers CK1α to the mitotic spindle to ensure proper spindle orientation and timely mitotic progression ^28^. The PAWS1-CK1α complex appears to regulate Wnt signalling by controlling the nuclear accumulation of β-catenin downstream of the β-catenin destruction complex through as-yet-unknown mechanisms ^1^. Establishing PAWS1-dependent CK1α substrates involved in mediating Wnt signalling will shed light on the mechanisms by which the pathogenic PPK PAWS1 mutants malfunction in Wnt signalling.

Despite the diverse functions of CK1α and associated FAM83 complexes, it is interesting to note that the ablation of CK1α from keratinocytes in mice resulted in palmoplantar and hair phenotypes similar to those associated with the two PAWS1 mutations ^29^. Although these phenotypes were not characterised in detail, it would be interesting to compare them morphologically and at the molecular level with those from PAWS1-mutant PPK phenotypes from human patients and dogs. As chemical inhibitors of CK1 isoforms are known to affect Wnt ^30– 34^ and p53 signalling ^35^, it is not surprising that complete ablation of CK1α from keratinocytes leads to the activation of both Wnt and p53 signalling. We suggest that differences in the relative levels of Wnt signalling components that are positively or negatively regulated by CK1α between cell types/tissues may ultimately determine the phenotype caused by PAWS1-CK1α dysregulation. We also cannot rule out the contributions of other signalling pathways known to be active in skin, including, but not limited to, TGF-β/BMP, FGF, and YAP/TAZ ^12, 36– 38^. Given that PAWS1 is highly expressed in the epidermal layer and inner root sheath of hair follicles ^9, 39^, it may be that PAWS1 is required for tissue-specific regulation of CK1α activity and modulation of signalling responses in these compartments. Characterisation of PAWS1 function in animal models or skin organotypic cultures will hopefully provide more definitive evidence of this in the future.

Finally, we also report here that PAWS1 mutant proteins have significantly shorter half-lives in cells. Consistent with these findings, immunostaining of skin sections revealed reduced levels of PAWS1 ^A34E^ protein in a patient suffering from PPK ^9^. Computational structure predictions suggest that residue A34 is positioned at the centre of an alpha helix, and R52 at the amino-terminal boundary of the following alpha helix ^40, 41^. Taken together with the radical nature of the A-E and R-P amino acid substitutions—hydrophobic to negative/hydrophilic, and positive/hydrophilic to hydrophobic respectively—it is likely that PAWS1 ^A34E^ and PAWS1 ^R52P^ are misfolded and subsequently degraded in a proteasome-dependent manner. Determination of the structure of PAWS1 or DUF1669 in complex with CK1α will allow accurate mapping of the residues that directly form the interface, and, given the high degree of conservation of the DUF1669, will no doubt be invaluable in understanding broader aspects of FAM83 and CK1 kinase biology.

## Data availability

### Underlying data

Open Science Framework: Pathogenic FAM83G palmoplantar keratoderma mutations inhibit the PAWS1:CK1α association and attenuate Wnt signalling. https://doi.org/10.17605/OSF.IO/FBQWY ^18^

This project contains the following underlying data

- Immunoblots
  ◦ Figure 1.pptx (PowerPoint file containing raw immunoblot images for Figure 1)
  ◦ Figure 2.pptx (PowerPoint file containing raw immunoblot images for Figure 2)
  ◦ Figure 3.pptx (PowerPoint file containing raw immunoblot images for Figure 3)
  ◦ Figure 4.pptx (PowerPoint file containing raw immunoblot images for Figure 4)
  ◦ Figure 5.pptx (PowerPoint file containing raw immunoblot images for Figure 5)

- Luciferase assay
  ◦ Figure 3B.xlsx (Excel spreadsheet containing luciferase assay data presented in Figure 3B)

- qPCR
  ◦ Figure 4.xlsx (Excel spreadsheet containing raw Ct values for qPCR)

- DNA sequencing
  ◦ 12-HGKO-B19-M13 Fwd-150617-12-56.ab1 (Sequence trace for HaCaT PAWS1 KO, Allele 1)
  ◦ 16-HGKO-B19-M13 Fwd-130617-10-34.ab1 (Sequence trace for HaCaT PAWS1 KO, Allele 2)
  ◦ 21-HGKO-B19-M13 Fwd-130617-10-39.ab1 (Sequence trace for HaCaT PAWS1 KO, Allele 3)
  ◦ A34E C3-7-M13 Fwd-231018-01-29.ab1 (Sequence trace for U2OS PAWS1 A34E Knock-in)
  ◦ UGKO_KW_14-M13 Fwd-160718-01-14.ab1 (Sequence trace for U2OS PAWS1 KO, Allele 1)
  ◦ UGKO_KW_16-M13 Fwd-160718-01-16.ab1 (Sequence trace for U2OS PAWS1 KO, Allele 2)
  ◦ UGKO_KW_24-M13 Fwd-160718-01-24.ab1 (Sequence trace for U2OS PAWS1 KO, Allele 3)

- Coomassie
  ◦ Figure 1E – SDS-PAGE Coomassie.pptx (PowerPoint file containing raw Coomassie stained gel image)

- Supplementary
  ◦ Fig_S2_Immunofluorescence.zip (Raw DeltaVision .dv image files for Figure S2)
  ◦ Figure S3 – Immunoblots.pptx (PowerPoint file containing uncropped blots)
  ◦ Figure S4 - qPCR.xlsx (Excel spreadsheet containing raw Ct values for qPCR)
  ◦ Figure S5 – Immunoblots.pdf (PDF containing uncropped blots)
  ◦ Figure S6 – DNA agarose gel.pptx (PowerPoint file containing raw agarose gel image for Figure S4)

- Mass spectrometry
  ◦ KWu 181203.sf3 (Scaffold file of mass spectrometry data shown in Figure 1E–G)

- Flow cytometry
  ◦ Flow cytometry.pptx (Flow cytometry plots showing gating strategy for single cell sorting of PAWS1 KO and A34E KI CRISPR clones)
  ◦ U2OS A34E KI.fcs (Raw output file for A34E KI sort)
  ◦ U2OS WT control.fcs (Raw output file for GFP negative population used as the control for the A34E KI sort)
  ◦ U2OS PAWS1 KO.fcs (Raw output file for single cell sort)

### Extended data

Open Science Framework: Pathogenic FAM83G palmoplantar keratoderma mutations inhibit the PAWS1:CK1α association and attenuate Wnt signalling. https://doi.org/10.17605/OSF.IO/FBQWY ^18^

This project contains the following extended data:

- Supplementary
  ◦ Wu *et al.* Supplementary.pdf (PDF containing supplementary figures)

Data are available under the terms of the Creative Commons Attribution 4.0 International license (CC-BY 4.0).
