# Supplementary material for: Pathogenic FAM83G palmoplantar keratoderma mutations inhibit the PAWS1:CK1α association and attenuate Wnt signalling
Source: Wellcome Open Res. 2020 Feb 17;4:133. Originally published 2019 Sep 9. [Version 2] doi: 10.12688/wellcomeopenres.15403.2 (PMC6798324; doi:10.12688/wellcomeopenres.15403.2)
Supplement: Peer review history

# Peer review: Reviewer response for version 2

**DOI:** 10.21956/wellcomeopenres.17258.r37932

**Published:** 2020-03-11

**Authors:** Uwe Knippschild

**Affiliations:** 1 Department of General and Visceral Surgery, Surgery Center, Ulm University Hospital, Ulm, Germany

The authors have satisfactorily responded to my comments.

I confirm that I have read this submission and believe that I have an appropriate level of expertise to confirm that it is of an acceptable scientific standard.

# Peer review: Reviewer response for version 1

**DOI:** 10.21956/wellcomeopenres.16836.r36733

**Published:** 2019-10-16

**Authors:** Patrick A. Eyers

**Affiliations:** 1 Department of Biochemistry, Institute of Integrative Biology, University of Liverpool, Liverpool, UK

This well-written research article comes from Wu and colleagues, and builds on some of their own earlier ground-breaking work. It helps us understand more about fundamental (classical) work looking at canonical Wnt signalling in human cells and in *Xenopus laevis* embryos. It also reveals very interesting biological effects of disease-associated FAM83G mutations in vertebrates; there is a hint at the end that these mutations might function through a common mechanism in other FAM83 genes also.

 The work brings together the recent genetic data from dog and human FAM83G/PAWS1 mutations (Ref 9), which are associated with keratoderma and excess hair. The study is essentially complete, is high quality, has an excellent Materials and Methods section, and raises lots of interesting questions for the future that will be useful for various groups interested in CK1, other FAM83 gene members and broader Wnt and CK1-dependent signalling mechanisms.

 The major output is to evaluate the interesting, potentially disruptive point mutations in the PAWS1/FAM83G 'DUF' (more likely CK1-binding) domain (R52P and A34E) by putting them through their paces in the context of CK1 and CD2AP/SMAD1 binding, broader proteomic interactomes, FAM83G cellular stability in several cellular models, axis duplication in frog embryos in vivo and also through generation of a very useful panel of knock-out/A34E knock-in lines. 

 I do not have any specific criticisms, the work is of a high quality, well explained and (most importantly) is very well controlled (often using previously characterized D262A/F296A FAM83G/PAWS1 mutants), and comes to sensible conclusions, clearly implicating the conserved Ala residue in the DUF domain of other FAM83 members as being involved in CK1 isoform targeting.   

Discussion point:

 The reduced stability of A34E and R52P FAM83G might have been compared side-by-side/or titrated at the mRNA level with D262A/F296A (or similar) in the Xenopus model, since the lack of phenotype here could, in theory, be explained by the decreased amount of FAM83G protein present (certainly <1/2 of the WT). The F296A is also 'more' stable (based on data in Figure 1C/E/G, all other things being equal). Obviously, it will be interesting to learn more about FA83G turnover, and how this might be controlled by proteome interactions (including CK1, but perhaps not through phosphorylation?). I would also purify recombinant FAM83G and the various mutants, and analyse whether they do indeed exhibit different thermal or chemical stability, or if the change in half-life is actually caused by the huge changes in partner interactions, or changes in PTMs. In this context, the clear difference between band-shifting of all the PAWS1 mutants tested in Figure 1C/D (HACAT KO cells) is distinct from the lack of change in shifting in Figure 1A/B (HEK-293), where the endogenous PAWS1 is presumably present.

I confirm that I have read this submission and believe that I have an appropriate level of expertise to confirm that it is of an acceptable scientific standard.

# Peer review: Reviewer response for version 1

**DOI:** 10.21956/wellcomeopenres.16836.r36462

**Published:** 2019-10-16

**Authors:** Uwe Knippschild

**Affiliations:** 1 Department of General and Visceral Surgery, Surgery Center, Ulm University Hospital, Ulm, Germany

In this study Wu and co-workers present data, clearly showing that pathogenic FAM83G palmoplanar keratoderma mutations either leading to the expression of PAWS1 ^A34E^ or PAWS1 ^R52P^ with reduced half-life compared to wt PAWS1. Both PAWS1 mutants are unable to interact with CK1a finally resulting in attenuation of Wnt signalling.

 The manuscript is well written, provides very interesting and novel findings regarding mechanisms involved in the regulation CK1a, a central player in Wnt signalling. Considering the importance of the Wnt pathway in regulating developmental processes, understanding of the functional consequences of PAWS1-CK1a interaction is of general interest.

 Overall, the work is carefully performed and clearly structured. Relevant literature is cited and the experimental part and data are sufficiently documented. However, the following few points should be addressed/discussed:

The authors state *“(i)…we consistently observed a lower abundance of  PAWS1 ^A34E^ and PAWS1 ^R52P^ proteins compared with PAWS1 ^WT^.”*
  The experiments performed provide clear evidence that stability of mutant proteins are abolished. However, pulse-chase experiments could further underline the conclusions drawn by the authors.
*(ii) “Furthermore, the mutant proteins had a lower apparent molecular weight on SDS PAGE compared with the WT protein. This suggests that the A34E and R52P might affect the stability of PAWS1 protein.”*
  Differences in molecular weight are often due to post-translational modifications, among them site-specific phosphorylation. Although the authors provide evidence that PAWS1 protein is not phosphorylated by CK1, it could still be phosphorylated by other kinases. To proof this assumption, PAWS1 WT could be separated by SDS PAGE after phosphatase treatment assuming that phosphate treatment results in conformational changes and faster migration of PAWS1 in SDS PAGE.
In the discussion the authors write “As chemical inhibitors of CK1 isoforms are known to affect Wnt and p53 signalling,”. The following citation could be added: García-Reyes *et al.* (2018 ^1^).
Finally, the authors suggest that structural analysis of PAWS1-CK1a complexes will allow accurate mapping of the residues that directly form the interface. In addition, the authors could discuss additional methods which could be used to determine the protein-protein interface which are described e.g. in Dolde *et al.* (2018 ^2^), Harnos *et al.* (2018 ^3^), Kruger *et al.* (2016 ^4^) and Huart *et al.* (2012 ^5^).

I confirm that I have read this submission and believe that I have an appropriate level of expertise to confirm that it is of an acceptable scientific standard.

## References

[1] : Discovery of Inhibitor of Wnt Production 2 (IWP-2) and Related Compounds As Selective ATP-Competitive Inhibitors of Casein Kinase 1 (CK1) δ/ε. *Journal of Medicinal Chemistry*.2018;61(9) : 10.1021/acs.jmedchem.8b00095 4087-4102 10.1021/acs.jmedchem.8b00095 29630366

[2] : A CK1 FRET biosensor reveals that DDX3X is an essential activator of CK1ε. *Journal of Cell Science*.2018;131(1) : 10.1242/jcs.207316 10.1242/jcs.207316 PMC581806029222110

[3] : Analysis of binding interfaces of the human scaffold protein AXIN1 by peptide microarrays. *Journal of Biological Chemistry*.2018;293(42) : 10.1074/jbc.RA118.005127 16337-16347 10.1074/jbc.RA118.005127 PMC620094330166345

[4] : New potential peptide therapeutics perturbing CK1δ/α-tubulin interaction. *Cancer Lett*.2016;375(2) : 10.1016/j.canlet.2016.03.021 375-383 10.1016/j.canlet.2016.03.021 26996302

[5] : Exploiting the MDM2-CK1α protein-protein interface to develop novel biologics that induce UBL-kinase-modification and inhibit cell growth. *PLoS One*.2012;7(8) : 10.1371/journal.pone.0043391 e43391 10.1371/journal.pone.0043391 22916255PMC3423359
